# Homœopathy and Pathology

**Published:** 1890-11

**Authors:** B. L. B. Baylies

**Affiliations:** Brooklyn, N. Y.


### HOMŒOPATHY AND PATHOLOGY.

B. L. B. Baylies, Brooklyn, N. Y.

The homœopathic physician is only successful and consistent with his profession when his practice is based upon a proper study of symptoms. A remedy's homœopathicity is not discernible in the pathologico-anatomical changes which its poisonous operation shows in the dead body; for other medicines may show a similar morbid anatomy: Phosphorus and Tartarized Antimony, for example, both cause inflammation of the brain, organs of respiration, stomach, and alimentary canal. Other medicines cause common inflammation of one or of several organs. How, then, shall a selection be made from several drugs of that one adapted to cure a particular case?

The old school prescribes, in a measure, according to some *a priori* theory of the pathological relation, but the homœpathician has verified the only law of cure, that discovered by Hahnemann, the only guide to the selection of the efficient remedy. Not according to presumed similarity of the anatomical changes caused by it to those *assumed* to exist in a given case, but according to that relation to the disease *process* determined by its similar symptoms, is its consequent eliminating and healing power. The nature, extent, and location of disease concern us for prognosis; and medicines may be partially classified for analytic and comparative study, according to their pathopoetic bearing upon the particular side or parts of the body affected; but pathological anatomy has no direct bearing on therapeutics, it relates to the not always possible *post-mortem* diagnosis, the detection of fatal poisoning, medical jurisprudence, rather than the art of healing. We are for healing purposes, operative surgery excepted, more interested in *subjective* than *objective* phenomena, whether presented immediately or mediately to our senses. And that mysterious, subtle process, which underlies all the phenomena, is the *true* and essential disease which only the homœopathic remedy is able to combat and annihilate.

